# Identification of the atypical cadherin FAT1 as a novel glypican-3 interacting protein in liver cancer cells

**DOI:** 10.1038/s41598-020-79524-3

**Published:** 2021-01-08

**Authors:** Panpan Meng, Yi-Fan Zhang, Wangli Zhang, Xin Chen, Tong Xu, Sheng Hu, Xinjun Liang, Mingqian Feng, Xiaoqing Yang, Mitchell Ho

**Affiliations:** 1 College of Life Science and Technology, Huazhong Agricultural University, No.1 Shizishan Street, Wuhan, 430070 Hubei Province China; 2 Laboratory of Molecular Biology, Center for Cancer Research, National Cancer Institute, National Institutes of Health, Bethesda, MD 20892 USA; 3 Hubei Cancer Hospital, Wuhan, 430079 Hubei China; 4 College of Biomedicine and Health, Huazhong Agricultural University, No.1 Shizishan Street, Wuhan, 430070 Hubei Province China; 5 Hospital of Huazhong Agricultural University, No.1 Shizishan Street, Wuhan, 430070 Hubei Province China

**Keywords:** Biochemistry, Cancer, Cell biology, Molecular biology, Gastroenterology

## Abstract

Glypican-3 (GPC3) is a cell surface heparan sulfate proteoglycan that is being evaluated as an emerging therapeutic target in hepatocellular carcinoma (HCC). GPC3 has been shown to interact with several extracellular signaling molecules, including Wnt, HGF, and Hedgehog. Here, we reported a cell surface transmembrane protein (FAT1) as a new GPC3 interacting protein. The GPC3 binding region on FAT1 was initially mapped to the C-terminal region (Q14517, residues 3662-4181), which covered a putative receptor tyrosine phosphatase (RTP)-like domain, a Laminin G-like domain, and five EGF-like domains. Fine mapping by ELISA and flow cytometry showed that the last four EGF-like domains (residues 4013-4181) contained a specific GPC3 binding site, whereas the RTP domain (residues 3662-3788) and the downstream Laminin G-2nd EGF-like region (residues 3829-4050) had non-specific GPC3 binding. In support of their interaction, GPC3 and FAT1 behaved concomitantly or at a similar pattern, e.g. having elevated expression in HCC cells, being up-regulated under hypoxia conditions, and being able to regulate the expression of EMT-related genes Snail, Vimentin, and E-Cadherin and promoting HCC cell migration. Taken together, our study provides the initial evidence for the novel mechanism of GPC3 and FAT1 in promoting HCC cell migration.

## Introduction

Liver cancer is the sixth most common cancer in terms of incidence and the fourth most common cancer-related death^1^. Primary liver cancer can be divided into hepatocellular carcinoma (HCC), cholangiocarcinoma (CCA), and mixed hepatocellular carcinoma. HCC is the most common form of primary liver cancer, accounting for about 90%^2^. Up to now, early diagnosis is the best way to control HCC. Four HCC biomarkers, alpha-fetoprotein (AFP), Golgi protein P73 (GP73), abnormal prothrombin (AP), and glypican-3 (GPC3), have been studied for early serological screening of HCC^3–6^. Further analysis is required to validate whether serum GPC3 can be used as a serological marker in HCC patients although cell surface GPC3 has been well established as a histochemical maker for HCC diagnosis^7,8^.

GPC3 is a heparan sulfate proteoglycan (HSPG), a member of the glypican family which contains six glypicans (GPC1-6)^7,9–11^. All glypicans have 14 conserved cysteine residues, 2 to 5 HS chains, and a glycosylphosphatidylinositol (GPI) anchor that attaches the glypicans to the cell membrane^7–9^. Glypicans are highly expressed during embryonic development, and some are specifically expressed in certain tissues in adults^11,12^. GPC3 has minimal and restricted expression in normal adult tissues such as lung, kidney, ovary, breast, and mesothelial tissues, but not liver^12^. As an oncofetal antigen, GPC3 is expressed in human embryos, participating in cell growth, differentiation, and morphogenesis^13–17^, and in HCC tissues, promoting tumor growth and migration^18–30^.

The Hippo/Yap pathway was initially discovered in *Drosophila*. The existence of this pathway was later confirmed in mammals^31^. The Hippo/Yap pathway acts through cell–cell contact inhibition^32^. The loss of contact inhibition is often seen in cancer; therefore, the Hippo/Yap pathway plays an important role in carcinogenesis. It has been established that the mammalian Hippo pathway is a potent regulator of liver organ growth, and that its dysregulation leads to liver tumorigenesis^31^. In previous studies, we found that an anti-GPC3 nanobody (HN3) or its fusion protein with a pseudomonas toxin could affect the Yap signaling in GPC3^+^ HCC tumor cells^19,22^. We found that the HN3 bound GPC3 and inhibited Yap signaling, indicating a functional association between GPC3 and Yap signaling in mammal cells. Yap is an independent prognostic marker of HCC^33^ and its overexpression can induce HCC in mouse model^31^. Studies in *Drosophila* indicate the role of a cell surface protocadherin (FAT) in Yap signaling during development^34^. Other studies indicate that FAT1 functions as the cell surface receptor of Yap signaling in mammalian cells^35–37^. However, how GPC3 might modulate Yap signaling is not clear.

FAT1 in humans is a large 506 kD type I transmembrane protein, consisting of 4588 amino acid residues, containing 34 Cadherin domains, a Laminin G domain, 5 EGF-like domains, a hydrophobic transmembrane (TM) region, and an intracellular domain (ICD)^38^. The intracellular domain of FAT1 binds to different proteins to regulate cell proliferation, migration, and polarity^39–41^. The FAT1 alterations occur in 29.8% of head and neck squamous cell carcinoma (HNSCC), and FAT1 functional loss results in YAP1 activation^36^. FAT1 is involved in the occurrence and development of cancers, functioning either as tumor suppressor or oncogenic gene depending on the cancer types^42^. Clinical data and studies have shown that FAT1 is a tumor suppressor in esophageal squamous cell carcinoma^43,44^, breast cancer^35^, and head and neck squamous cell carcinoma^36^. In contrast, FAT1 promotes tumorigenesis in acute lymphoblastic leukemia^45^, glioblastoma^46,47^, colon cancer^48,49^, gastric cancer^50^, oral squamous cell carcinomas^51,52^, and liver cancer^53^.

In the present study, we provided initial evidence showing that GPC3 interacts with FAT1. We also found that GPC3 and FAT1 express and function in similar patterns, including elevated expression induced by hypoxia and their role in promoting HCC cell migration.

## Materials and methods

### Cell lines and cell culture

The HCC cell lines HepG2, Hep3B, and Huh7 were cultured in DMEM medium (Gibco) supplemented with 1% (vol/vol) Ala-glutamine, 1% penicillin–streptomycin, and 10% FBS (HyClone), incubated in a 37 °C incubator with 5% CO_2_. Hypoxia was induced by incubation with 2,2-dipyridyl (DP) (100 µmol/L, A601219, Sangon Biotech) for the indicated periods of time.

### Antibodies

hYP7 is a humanized antibody that recognize a C-terminal epitope (residues 521–530) of GPC3 as previously described^30,54^. Other antibodies include anti-FAT1 (HPA023882, Sigma), anti-β-actin (66009-1-Ig, Proteintech), anti-HIF1α (20960-1-AP, Proteintech), and anti-FLAG (20543-1-AP, 66008-3-Ig, Proteintech).

### Preparation of recombinant GPC3 and FAT1 fragments

The coding sequence for GPC3 and FAT1 was based on GenBank accession number NM_004484.3 and NM_005245.3 respectively. The full-length GPC3 and truncated FAT1 sequences were cloned from the cDNA of Hep3B cells. According to different experimental conditions, a FLAG tag or hFc tag was added to the C-terminal end of FAT1 truncated fragments. The expression cassette was cloned into expression vector pFUSE and introduced into 293 T cells for expression by PEI transfection. FLAG-tagged FAT1 expressing 293 T lysate was used in the co-immunoprecipitation (co-IP) assay. Recombinant hFc-tagged protein was purified by using protein A affinity chromatography (Sangon Biotech).

### Protein extraction and western blot

Cells were cultured in flask or plate for indicated conditions, and lysed with RIPA buffer (P0013K, Beyotime) containing cocktail protease and phosphatase inhibitors (P1008, Beyotime). Protein concentration was measured by a BCA Protein Assay kit (P0011, Beyotime). Total proteins were separated on 10% reducing SDS PAGE gel and transferred to 0.2 μm PVDF membrane (Merck KGaA, Darmstadt, Germany) for Western blot analysis.

### Total RNA extraction and quantitative RT-PCR

Cells were cultured in flask or plate at indicated conditions, lysed with TRIzol (Cat.15596018, Invitrogen). Total RNA was extracted according to the manufacturer’s instructions. Three micrograms of total RNA were used in a reverse transcription reaction (Cat.28025021, ThermoFisher) according to the manufacturer’s instructions. Quantitative RT-PCR was performed using a qRT-PCR kit (RR820L, Takara) with specific primers as follow: FAT1-Forward, 5′-GCCTGTCTG.

AAGTGCAGTCT-3′; FAT1-Reverse, 5′-TGAGTACAGGGGTGTCTGCT-3′; GPC3-Forward, 5′-GAAAGTGGAGACTGCGGTGA-3′; GPC3-Reverse, 5′-TTGCCT

GCTGACTGTTTCCA-3′; HIF1α-Forward, 5′-GCCAGACGATCATGCAGCTA-3′;

HIF1α-Reverse, 5′-ATCCATTGATTGCCCCAGCA-3′; Snail-Forward, 5′-ACCTCCA

GACCCACTCAGAT-3′; Snail-Reverse, 5′-CCGGACTCTTGGTGCTTGT-3′; Vimentin-Forward, 5′-CACCTACAGGAAGCTGCTGG-3′; Vimentin-Reverse, 5′-AC

CAGAGGGAGTGAATCCAGA-3′; E-Cadherin-Forward, 5′-TCATGAGTGTCCCCC

GGTAT-3′; E-Cadherin-Reverse, 5′-GGGTCAGTATCAGCCGCTTT-3′.

### Knockdown of gene expression by shRNA

For gene knockdown, two pairs of shRNA-coding DNA oligos were synthesized and subsequently cloned into the shRNA expression vector pGreenPuro (System Biosciences) according to the manufacturer’s instruction. The control shRNA is a scrambled sequence that does not target any mammalian mRNA. The sequences of the shRNA target sites are indicated below.

GPC3sh1: 5′-GGAGCTCAAGTTCTTAATTAT-3′; GPC3sh2: 5′-ACTGCAAGTCAC

TAGGATCTT-3′; FAT1sh1: 5′-GGACCAGTATCGCAAGAGTCA-3′; FAT1sh2: 5′-G

AAGACAAGGAGGTACATAGT-3′; HIF1αsh1: 5′-GAATGAAGTGTACCCTAACT

A-3′; HIF1αsh2: 5′-GACGATCATGCAGCTACTACA-3′; shCtrl: 5′- GCGTAATAAC

GATGTCTCTAC-3′

For lentivirus packaging, the packing plasmids p-Mission-Gag/Pol and p-Mission-VSV-G were mixed with expression plasmid pGreenPuro in 1 ml of Opti-Mem medium at a ratio of 3:1:1, then threefold excess of PEI solution was mixed with the plasmid solution. After the plasmids and PEI formed complex at room temperature for 20 min, the plasmid-PEI mixture were transferred to a T-75 flask of 293 T cells, gently mixed and placed in the CO_2_ incubator for 3 days. The lentivirus-containing cell culture medium was collected and used to transduce target cells. Transduced cells were selected with 2 μg/ml of puromycin for 7 days to get stable knockdown cells.

### Cell migration assay

Cell migration was assessed by using Transwell plate inserts with 8 µm pores (Cat.3422, Costar) following the manufacturer’s instruction. Cells were harvested, counted, and added to the upper insert (2 × 10^4^/well). Fresh cell culture medium containing 20% FBS was added to the lower chamber. After 48 h, the upper chamber was washed and the un-migrated cells were removed. Cells were fixed with formaldehyde and stained with crystal violet. The staining was recorded by taking photographs and cell migration was calculated by comparison of the migrated cells between the control and treatment group.

### Co-IP

For the determination of the interaction between the endogenous GPC3 and FAT1, GPC3 from HepG2 cell lysate was pooled down with hYP7, and detection of endogenous FAT1 was visualized by HRP-conjugated anti-FAT1 antibody. Pooled human IgG (hIgG) was used as isotype control of hYP7.

To map the GPC3-binding region on FAT1, full-length GPC3 and FLAG-tagged FAT1 truncation fragments were co-expressed in 293 T cells. Cell lysate was immunoprecipitated by incubating 0.8 mg of total protein in RIPA buffer with 5 µg of the anti-FLAG antibody at 4 °C overnight. The mixture of the cell lysate and antibody was incubated with protein A/G Agarose (36403ES03, Yeasen) at room temperature for 1 h with gentle rotation. After 5 times of washing, the immune complex was recovered by boiling in SDS-PAGE loading buffer. FAT1 fragment-associated GPC3 was probed and visualized by western blot.

### ELISA

To measure the binding of GPC3 and various FAT1 fragments, recombinant His-tagged GPC3 was immobilized on a 96-well plate in PBS buffer at a final concentration of 5 μg/mL, and then blocked with 5% (w/v) BSA in PBS buffer. Various amounts of recombinant hFc-tagged FAT1 fragments was added to the plate, followed by incubation at 37 °C for 1 h to allow the binding to occur. Pooled hIgG (Cat.I4506, Sigma) was used as isotype control. After washing the plate twice with PBS buffer containing 0.05% Tween 20, the binding was detected by an HRP-conjugated goat-anti-human antibody (Cat.109-036-170, Jackson ImmunoResearch). The A_450_ values were associated with the corresponding FAT1 fragment concentration, and the EC_50_ values were determined by GraphPad Prism 5.0 software.

### Flow cytometry

To determine whether FAT1 was able to bind cell surface GPC3, flow cytometry method was used. GPC3-negative A431 and GPC3 over-expressing A431 (GPC3^+^) cells were used as tested cell lines. Cells were harvested, washed twice with PBS buffer, and resuspended in ice-cold PBS buffer containing 5% (w/v) BSA. One million of cells were incubated with 50 μg of purified FAT1-hFc fragment and hIgG isotype control (Cat.I4506, Sigma). Cell binding of FAT1-hFc fragment was detected by goat anti-human IgG conjugated with Alexa Fluor488 (ab150077, Abcam).

### Statistical analysis

Data analyses were performed by using the GraphPad software and expressed as the mean ± SEM. Comparisons of two groups were performed using Paired Student's t test (two-tailed). Comparisons among three or more groups were performed using one-way ANOVA. *P* value less than 0.05 was considered statistically significant.

## Results

### The interaction of GPC3 and FAT1

Given that GPC3 could modulate the Yap signaling, and FAT1 was suggested as the cell surface receptor of Yap signaling in mammalian cells, we postulated that GPC3 might interact with FAT1.

**Figure 1:**
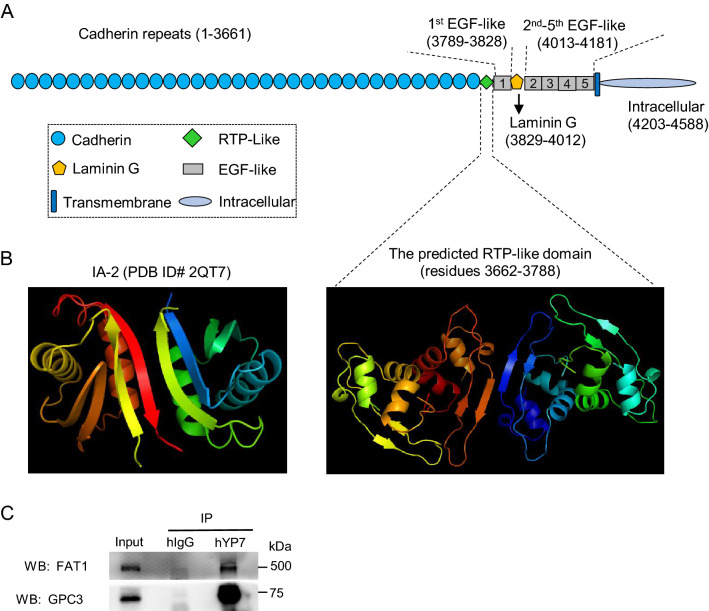
The structure of FAT1 and its co-IP with GPC3. (**A**) Schematic diagram of FAT1 structure. (**B**) Structural model of FAT1 (residues 3662-3788). The model was built on the crystal structure of the human receptor tyrosine phosphatase IA-2 (insulinoma- associated protein 2) (PDB ID# 2QT7), and FAT1 (residues 3662-3778) structure resembled the extracellular domain of IA-2. The 3D structural model was made by using the software SWISS-MODEL (https://swissmodel.expasy.org/). (**C**) co-IP of endogenous FAT1 by GPC3 in HepG2 cells. GPC3 was pulled down by the hYP7 antibody. The co-IP of FAT1 was detected by anti-FAT1 antibody. Pooled hIgG was used as isotype control of hYP7. The original blots were presented in Supplementary Figure S1.

FAT1 is an enormous protein that contains 34 cadherin domains (Fig. 1A). The region (residues 3662-3788) downstream of the last cadherin was not annotated, and its crystal structure has not been determined. To visualize the possible structure and function of this region, we modeled its 3D structure by using the web tool SWISS-MODEL (https://swissmodel.expasy.org/), which predicted that the overall structure resembled the extracellular domain of human receptor tyrosine phosphatase IA-2 (insulinoma-associated protein 2) (PDB ID# 2QT7), herein we tentatively named this region RTP domain (Fig. 1B). However, it is unclear whether this RTP domain has any phosphatase activity.

To testify our prediction about the interaction of GPC3 and FAT1, we conducted co-IP to analyze the interaction between the endogenous GPC3 and FAT1. As shown in Fig. 1C, endogenous FAT1 in HCC cell lysate could be pulled down by the hYP7 antibody specific for the C-terminal epitope of GPC3.

### Mapping of GPC3 binding region on FAT1

**Figure 2:**
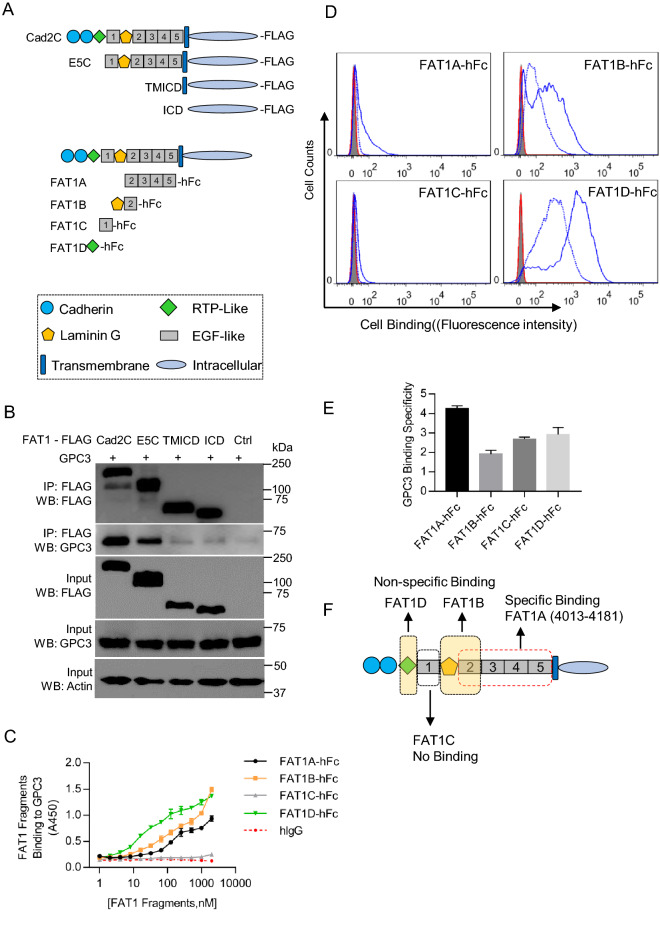
Mapping of the GPC3 binding region on FAT1. (**A**) Diagram of the FAT1 fragments, either with C-terminal FLAG tag for co-IP assays, or with C-terminal hFc tag for ELISA and FACS experiments. (**B**) Co-IP of GPC3 by FAT1 truncation fragments with C-terminal FLAG tag in 293 T cells. GPC3 and FAT1 truncation fragments were co-expressed in 293 T cells. FAT1 fragments were pulled down with anti-FLAG monoclonal antibody, and the co-immunoprecipitated GPC3 was probed with anti-GPC3 monoclonal antibody hYP7. Ctrl was an empty expression vector for FAT1 fragments. Full-length blots/gels were presented in Supplementary Figure S2. (**C**) ELISA analysis of recombinant FAT1 fragments binding to immobilized GPC3. GPC3-His was coated on the 96-well plate (5 μg/ml, 50 μl/well), and varied amount of FAT1 fragments was added to the wells. Pooled hIgG was used as isotype control. (**D**) Flow cytometry analysis of the binding specificity of the recombinant FAT1 fragments to cell surface GPC3. A pair of GPC3 negative A431 cell line and GPC3 over-expressing A431 (GPC3+) cells were tested by incubation with hFc-tagged FAT1 fragments (50 μg/ml). Blue solid line, FAT1 fragments staining on A431 (GPC3+) cells; Blue dashed line, FAT1 fragments staining on A431; red line, isotype control staining on A431 (GPC3+); Shaded area, isotype control staining on A431. (**E**) Binding specificity of recombinant FAT1 fragments to cell surface GPC3 that was calculated by the ratio of A431 (GPC3+) binding (geometrical mean of fluorescence intensity) versus A431, based on data from (**D**). Data represent mean ± SEM (n = 3). (**F**) Schematic diagram of the GPC3 binding region on FAT1 based on the above data.

To map the GPC3 binding region on FAT1, we constructed a series of N-terminal truncated fragments fused with FLAG-tag (Fig. 2A). The FLAG-tagged FAT1 fragments were co-expressed with the full-length GPC3 in 293 T cells. We conducted co-IP assay to examine their interactions. As shown in Fig. 2B, we found that fragments Cad2C (covering the last two cadherin domains and the downstream whole region) and E5C (covering 1st EGF-like domain and the downstream whole region) retained the ability to co-IP GPC3, while the transmembrane-intracellular region (TMICD) and the intracellular domain (ICD) did not pull down GPC3, indicating the extracellular region proximal to the C-terminus of FAT1 has GPC3 binding domain.

To further narrow down the GPC3 binding region, we expressed and purified smaller FAT1 fragments as hFc fusions (named FAT1A to D) such that each fragment contains the different type of FAT1 functional or structural domains (Fig. 2A). Fragment FAT1A covered the last four EGF-like domains, FAT1B covered the Laminin G and the 2nd EGF-like domain, FAT1C corresponded to the 1st EGF-like domain, and FAT1D corresponded to the putative RTP domain. Protein binding ELISA showed that FAT1B and FAT1D had strong GPC3 binding (Fig. 2C). FAT1A had relative weaker GPC3 binding. FAT1C had no GPC3 binding. The bind data indicates that the RTP, Laminin G, and the last four EGF-like domain may contain the GPC3 binding region.

To check the GPC3 binding specificity, flow cytometry was performed to measure the specific binding of the above-mentioned domains to GPC3 that was artificially expressed on the cell surface of GPC3-negative A431 cells. The ratio of A431 (GPC3^+^) binding versus A431 was calculated based on the geometrical mean of fluorescence intensity and was used to indicate the binding specificity of each domain (Fig. 2D,E). The results showed that FAT1A had the highest binding specificity to GPC3, with very low levels of non-specific binding to A431 cells. However, FAT1B and FAT1D had strong but non-specific binding to A431 cells to some degree, especially FAT1D. FAT1C showed neglectable binding on either A431 (GPC3+) cells or A431 cells, which is consistent with protein binding in Fig. 2C. Taken together, it clearly showed that FAT1A (the last four EGF-like domains) had the specific GPC3 binding region as summarized in Fig. 2F.

### Elevated expression of GPC3 and FAT1 in HCC cell lines

**Figure 3:**
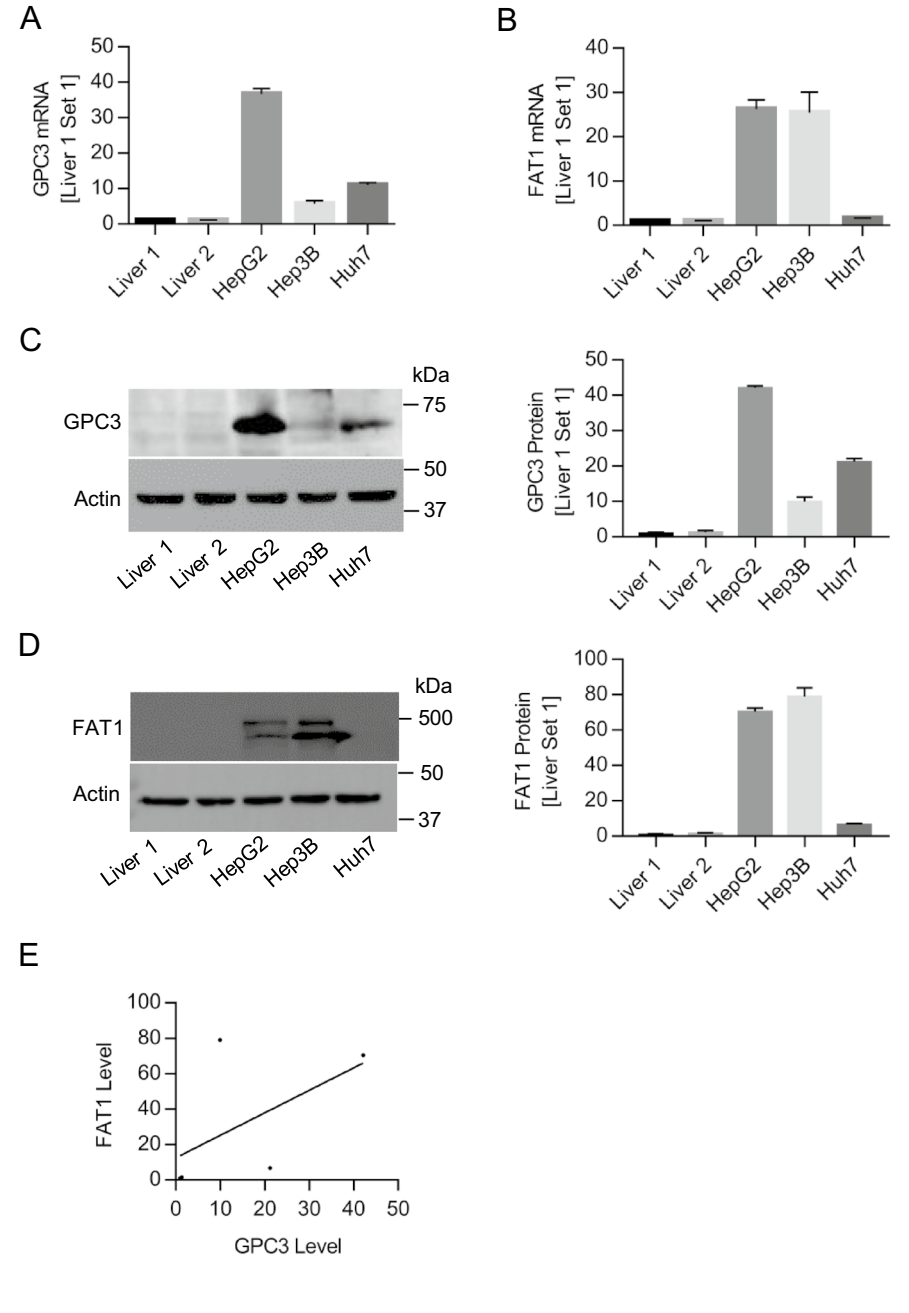
Elevated expression of GPC3 and FAT1 in HCC cell lines. (**A**) The mRNA level of GPC3 in two primary liver tissues and HCC cell lines (HepG2, Hep3B and Huh7) measured by quantitative RT-PCR. (**B**) The mRNA level of FAT1 in the same samples. (**C**) GPC3 protein level of the corresponding tissues and cell lines. Full-length blots/gels were presented in Supplementary Figure S3A. (**D**) FAT1 protein level from the same samples. Full-length blots/gels were presented in Supplementary Figure S3B (**E**) Correlation of GPC3 and FAT1 protein level in three HCC cell lines (HepG2, Hep3B and Huh7). Data represent mean ± SEM (n = 3).

It was known both GPC3 and FAT is a proto-oncogene or tumorigenic gene in HCC, with low or neglectable expression in normal adult liver^3,53^. We compared the expression pattern of GPC3 and FAT1 in normal adult liver tissues and HCC cell lines by quantitative real-time PCR and Western blot (Fig. 3). The expression of GPC3 and FAT1 in two normal adult liver tissues could barely be detected at both mRNA (Fig. 3A,B) and protein level (Fig. 3C,D), but appeared very high on HCC cell lines. We tried to correlate the protein level of GPC3 and FAT1 in three HCC cell lines, and it seemed that GPC3 and FAT1 had a co-expression trend in HCC cells, with a R^2^ value of 0.32 (Fig. 3E), implying the correlation of the two proteins.

### Up-regulated expression of HIF1α, GPC3, and FAT1 in hypoxia settings

**Figure 4:**
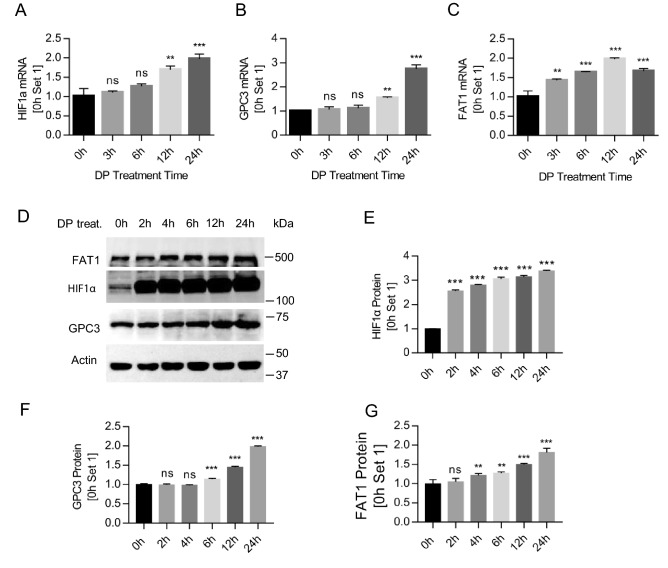
Up-regulated expression of HIF1α, GPC3, and FAT1 under hypoxia. (**A**) HIF1α mRNA level in HepG2 cells after DP (100 µM) treatment for different lengths of time. (**B**) GPC3 and (**C**) FAT1 mRNA levels after same treatment as (**A**). (**D**) Western blot analysis of HIF1α, GPC3, and FAT1 protein levels in HepG2 cells after DP (100 µM) treatment. Full-length blots/gels were presented in Supplementary Figure S4. Quantification was performed based on the band intensity and presented in (**E**) HIF1α, (**F**) GPC3, and (**G**) FAT1. Data represent mean ± SEM (n = 3). **P < 0.01; ***P < 0.001. ns, not significant.

HCC development and progression are regulated by the tumor microenvironment. Hypoxia is one of the major features of the tumor microenvironment^55^, and HIF1α plays an important role in hypoxia and tumorigenicity^56,57^. To gain insight into the expression pattern of GPC3 and FAT1 under hypoxia conditions, we treated HepG2 cells with chemical hypoxia inducer 2,2-dipyridyl (DP) as previously described^53^. As shown in Fig. 4, DP treatment significantly up-regulated the expression of HIF1α, GPC3, and FAT1 at both mRNA (Fig. 4A–C) and protein level (Fig. 4D–G). HIF1α protein level responded quickly to hypoxia induction, with an abrupt rising in 2 h of treatment. The up-regulation of GPC3 and FAT1 by DP treatment was apparently lagging behind that of HIF1α.

### Involvement of GPC3 and FAT1 in HCC cell migration

**Figure 5:**
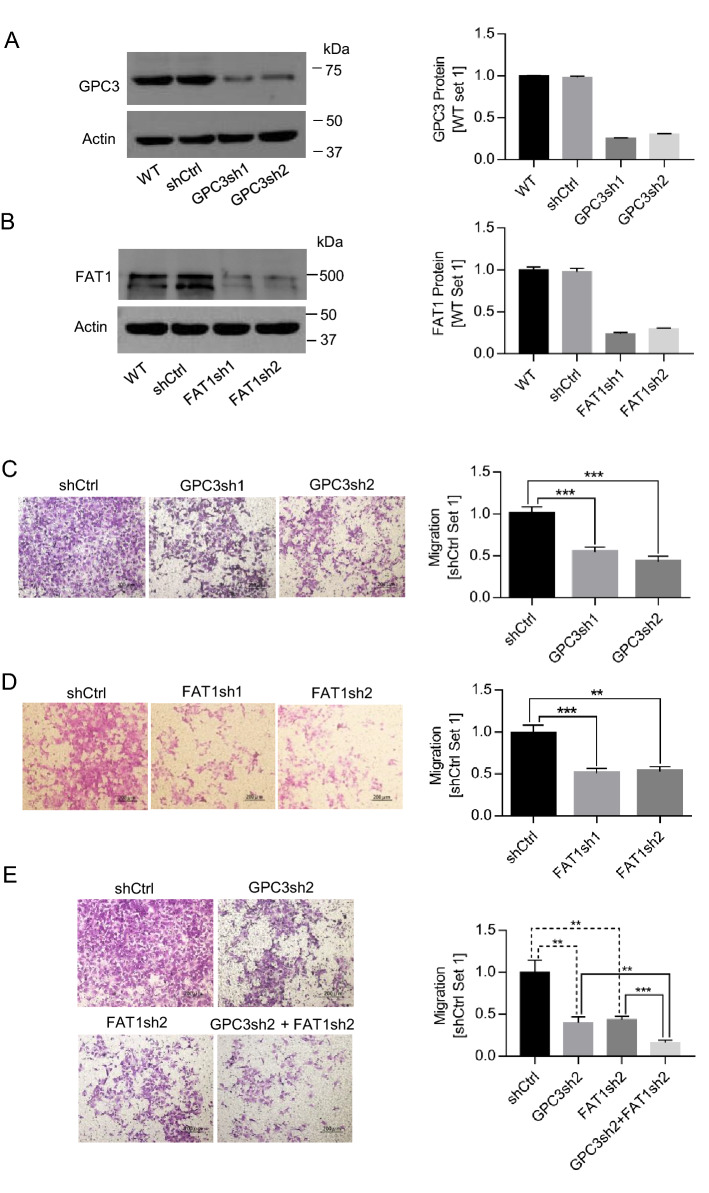
Involvement of GPC3 and FAT1 in HCC cell migration. (**A**) Knock-down of GPC3 in Hep3B cells by stably transfection with GPC3 specific shRNA (GPC3sh1 and GPC3sh2) or control shRNA (shCtrl). Full-length blots/gels were presented in Supplementary Figure S5A. (**B**) FAT1 knock-down. Full-length blots/gels were presented in Supplementary Figure S5B. (**C**) Migration of Hep3B cells after GPC3 knock-down. Migration assays were performed by using Transwell method. The staining of migrated cells was recorded by taking photographs and cell migration was calculated by the difference of migrated cells between the control and treatment group (n = 3/group). (**D**) Migration of Hep3B cells after FAT1 knock-down. (**E**) Migration of Hep3B cells after GPC3 and FAT1 double knock-down. Data represent mean ± SEM (n = 3). **P < 0.01; ***P < 0.001.

Metastasis is a major attribute of cancer aggressiveness. In order to clarify the roles of GPC3 and FAT1 in HCC cell migration, we knocked down GPC3 and FAT1 expression by two shRNAs in Hep3B cells, as confirmed by Western blot (Fig. 5A,B). The effect of GPC3 and FAT1 knockdown on cell migration was determined by Transwell assay. Compared with shCtrl, both GPC3 and FAT1 knockdown significantly inhibited cell migration (Fig. 5C,D). GPC3 and FAT1 single knock down had no significant difference in suppressing cell migration, while double knock down had additive effect (Fig. 5E), indicating that the direct association of GPC3 and FAT1 might form a functional complex in suppressing migration.

### PC3 and FAT1 regulated the expression of EMT related genes

**Figure 6:**
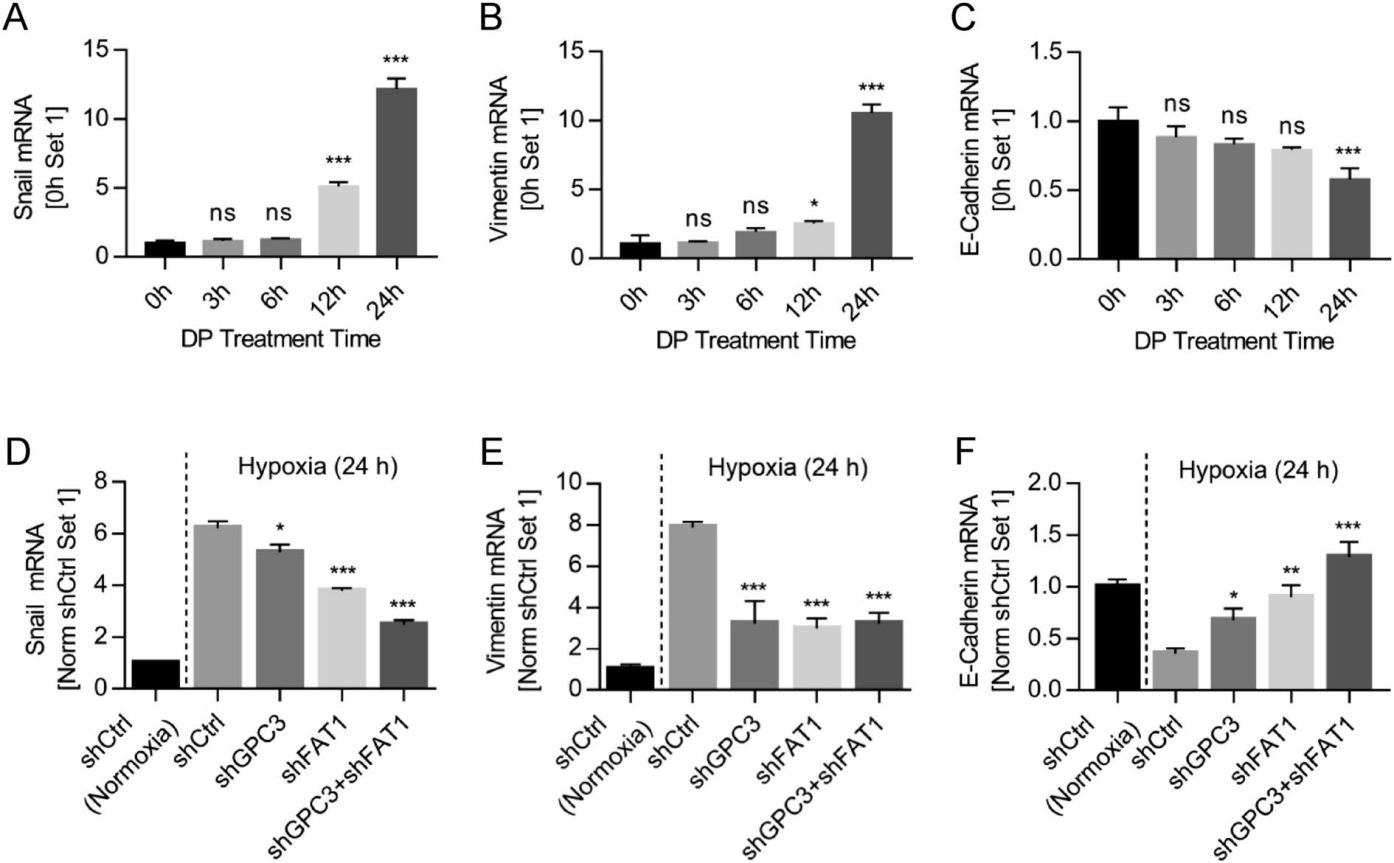
Expression of EMT related genes regulated by GPC3 and FAT1. The expression of hypoxia-regulated genes (**A**) Snail, (**B**) Vimentin, and (**C**) E-Cadherin was studied by quantitative RT-PCR in HepG2 cells after DP (100 µM) treatment. The impact of GPC3 and FAT1 knockdown on the expression of (**D**) Snail, (**E**) Vimentin, and (**F**) E-Cadherin was investigated under DP-simulated hypoxia condition. Data represent mean ± SEM (n = 3). *P < 0.05; **P < 0.01; ***P < 0.001. ns, not significant.

To mechanistically understand the interaction of GPC3 and FAT1 in promoting HCC cell migration, we analyzed the regulation pattern of GPC3 and FAT1 on the expression of tumor metastasis-related genes under hypoxic conditions. As shown in Fig. 6, when HepG2 cells were treated with DP for 3 to 24 h, the expression of Snail and Vimentin was gradually up-regulated, and E-Cadherin was gradually down-regulated (Fig. 6A–C). Knockdown of GPC3 and FAT1 alone or in combination suppressed the DP-induced up-regulation of Snail and Vimentin, and down-regulation of E-Cadherin (Fig. 6D–F). Double knockdown had additive effect in the suppression of Snail and E-Cadherin (Fig. 6D,F). In the case of Vimentin, single knockdown of either GPC3 or FAT1 reached the maximal suppression (Fig. 6E), suggesting again that GPC3 and FAT1 may function as a complex.

## Discussion

Previous studies demonstrated that GPC3 promoted HCC cell migration by recruiting extracellular Wnt and HGF factors and transferring them to the corresponding receptors^23,58^. We have also demonstrated that GPC3 is involved in Yap signaling^19,22^. Since GPC3 does not have intracellular signaling domain, we postulated that GPC3 may also directly interact with some receptor-like transmembrane protein to transmit the signal. In the present study, we identified the atypical cadherin FAT1 as a new GPC3-interacting protein.

To map the GPC3 binding region on FAT1, we first focused on the functional domains, the cadherin domains, the RTP-like domain, the first EGF-like domain, the Laminin G domain, and the last four EGF-like domains, all of which are located toward the C-terminus of FAT1 extracellular region. For this purpose, the N-terminal shortened fragments that retained all or some of the functional domains were co-expressed with GPC3 in 293 T cells, and the subsequent co-IP assays determined that the cadherin repeats and the RTP-like domain may not be involved in GPC3 binding (Fig. 2B). To further identify the GPC3 binding domain on FAT1, four recombinant FAT1 fragments were expressed and purified (Fig. 2A). The protein binding ELISA assay showed that FAT1D (the RTP domain), FAT1B (the Laminin G and the first EGF-like domain), and the FAT1A region (the last four EGF-like domains) were able to bind GPC3 (Fig. 2C), FAT1C (the first EGF-like domain alone) had no GPC3 binding. To evaluate the binding specificity on cells, we over-expressed GPC3 on A431 cells. The specific binding of FAT1 domains on A431 (GPC3+) versus A431 was compared. It showed that FAT1A had the most specific binding to A431 (GPC3), while FAT1B had less specific binding to GPC3 since it also bound to A431 cells. The FAT1C did not bind to the cell, which is consistent with our ELISA data. The FAT1D had no specificity for GPC3 binding because it bound to A431 (GPC3) equally strong as A431 (Fig. 2D,E). Taken together, it is clear that the last four EGF-like domains represented by FAT1A (residues 4013-4181) on FAT1 have the specific GPC3 binding domain (Fig. 2F). Laminin G and the RTP domain could bind GPC3 on cells nonspecifically. Interestingly, the first EGF-like domain as represented in FAT1C did not bind GPC3.

In view of previous studies, both GPC3 and FAT1 are highly expressed in HCC and correlated with poor prognosis, and promote HCC cell migration and proliferation^24,53,59,60^. Here, we found that high-level expression of GPC3 coincided with that of FAT1 in all the tested liver cancer cell lines (HepG2, Hep3B, and Huh7), and undetectable in normal liver tissues (Fig. 3). Moreover, impaired GPC3 and FAT1 expression by shRNA knockdown suppressed HCC cell migration in a comparable level (Fig. 5), although double knockdown of GPC3 and FAT1 had a slightly greater inhibition of HCC cell migration compared to single knockdown (Fig. 5). Taken these data with previous findings that both GPC3 and FAT1 were able to modulate the activity of YAP, the downstream effector of Hippo pathway^35,61^, it would be reasonable to postulate that GPC3 and FAT1 might function as complex in promoting HCC cell migration.

Hypoxia is a major feature of tumor microenvironment and a driving force to promote cancer metastasis, including HCC^55,62–64^. Hypoxia induces the expression of a number of genes^65–67^, and some of them drive tumor metastasis, e.g. HIF1α^68^. FAT1 is another important gene that regulates EMT and stemness characteristics in hypoxia GBM tumor^43^ and drives tumor metastasis^53^. The current study used DP treatment to simulate hypoxia environment as previously described^53^, and it was found that DP treatment significantly up-regulated the expression of HIF1α, GPC3, FAT1, and other tumor metastasis-related genes (Snail, Vimentin) (Figs. 4,6). Furthermore, both GPC3 and FAT1 regulated the expression of tumor metastasis-related genes, e.g., Snail, Vimentin, and E-Cadherin (Fig. 6D–F). As expected, single knockdown of either GPC3 or FAT1 suppressed the regulation of metastasis genes Snail, Vimentin, and E-Cadherin, and double knockdown of GPC3 and FAT1 had a little enhanced effect on the regulation of some of the EMT genes (Fig. 6), suggesting again that GPC3 and FAT1 may work via a shared mechanism to promote HCC cell migration.

In conclusion, the current work identified the direction interaction of transmembrane protein FAT1 and GPC3. In support of this observation, FAT1 and GPC3 had similar expression patterns and functional features in terms of promoting HCC cell migration, and both GPC3 and FAT1 could regulate the expression of metastasis-related genes.

## Supplementary Information

Supplementary Figures.

## Data Availability

Data sharing is not applicable to this article as no datasets were generated or analyzed during the current study. Materials from the present study are available from the corresponding author on reasonable request.
