# Supplementary material for: Identification of the atypical cadherin FAT1 as a novel glypican-3 interacting protein in liver cancer cells
Source: Sci Rep. 2021 Jan 8;11:40. doi: 10.1038/s41598-020-79524-3 (PMC7794441; doi:10.1038/s41598-020-79524-3)
Supplement: Supplementary file 1 — Supplementary Figures. [file 41598_2020_79524_MOESM1_ESM.doc]

Supplementary Information

Identification of the atypical cadherin FAT1 as a novel glypican-3 interacting protein in liver cancer cells

Panpan Meng^1^, Yi-Fan Zhang^2^, Wangli Zhang^1^, Xin Chen^1^, Tong Xu^1^, Sheng Hu^3^, Xinjun Liang^3^, Mingqian Feng^4*^, Xiaoqing Yang^5*^, Mitchell Ho^2*^

^1^College of Life Science & Technology, Huazhong Agricultural University, No.1 Shizishan Street, Wuhan, Hubei Province 430070, China;

^2^Laboratory of Molecular Biology, Center for Cancer Research, National Cancer Institute, National Institutes of Health, Bethesda, Maryland 20892, USA;

^3^Hubei Cancer Hospital, Wuhan, Hubei 430079, China;

^4^College of Biomedicine and Health, Huazhong Agricultural University, No.1 Shizishan Street, Wuhan, Hubei Province 430070, China;

^5^Hospital of Huazhong Agricultural University, No.1 Shizishan Street, Wuhan, Hubei Province 430070, China;

^*^**Corresponding to:**

Fengmingqian@mail.hzau.edu.cn; Yangxiaoqing@mail.hzau.edu.cn; Homi@mail.nih.gov

**Key words：**GPC3, FAT1, Yap, Hepatocellular carcinoma, cell migration

Supplementary Figures

**
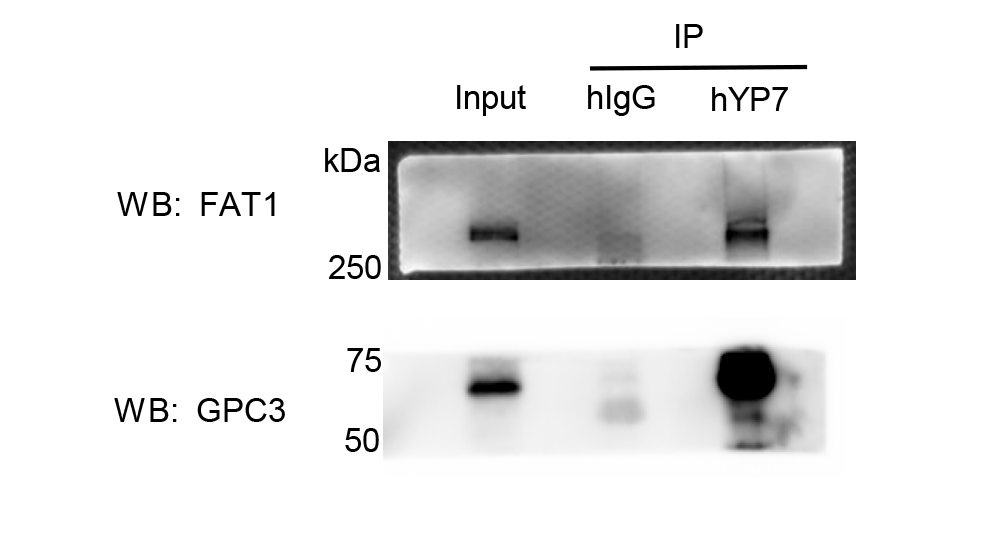
**

**Fig. S1.** Co-IP of endogenous FAT1 with GPC3 in HepG2 cells (**Fig. 1C** in the main text). To measure the relative quantity of the co-precipitated FAT1 along with that of the precipitated GPC3 by the anti-GPC3 beads, samples were run on the same gel and cut into two parts with each stained for the target band of FAT1 or GPC3.

**
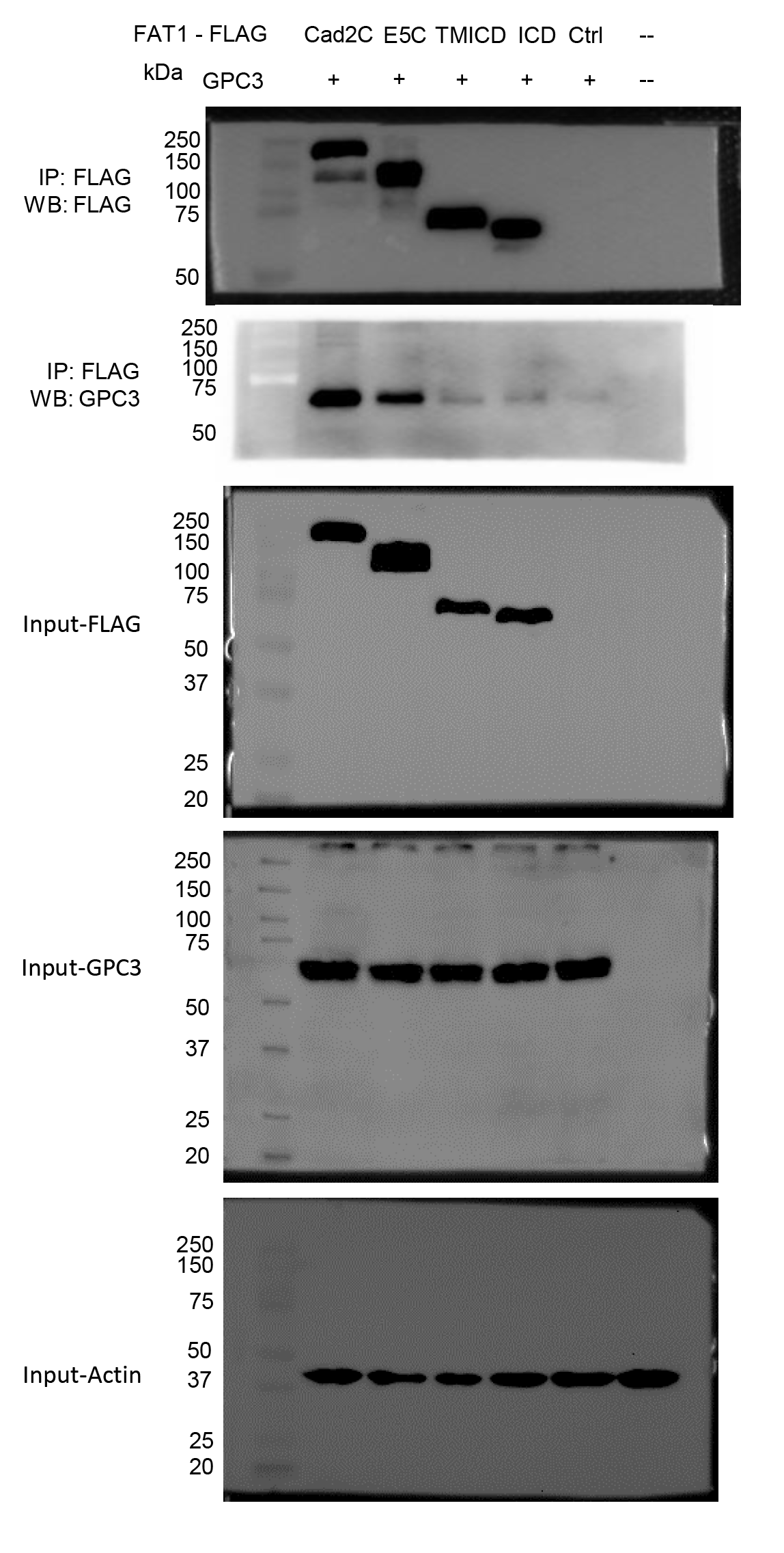
**

**Fig. S2.** Co-IP of GPC3 by FAT1 truncation fragments with C-terminal FLAG tag in 293T cells (**Fig. 2B** in the main text).

GPC3 and FAT1 truncation fragments were co-expressed in 293T cells. FAT1 fragments were pulled down with an anti-FLAG monoclonal antibody, and the co-immunoprecipitated GPC3 was probed with anti-GPC3 monoclonal antibody hYP7. Ctrl (control) was an empty expression vector for FAT1 fragments.

**Fig. S3**

**
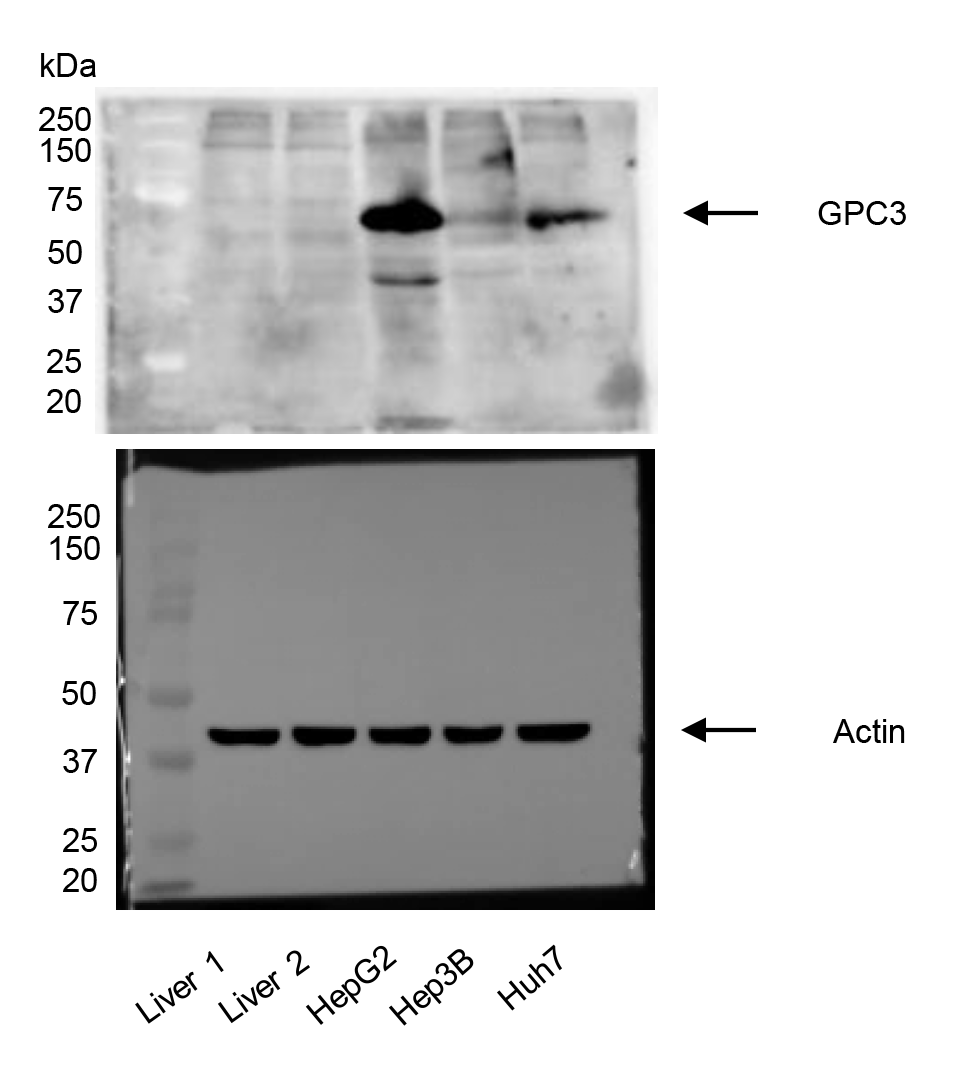
**

**A**

**
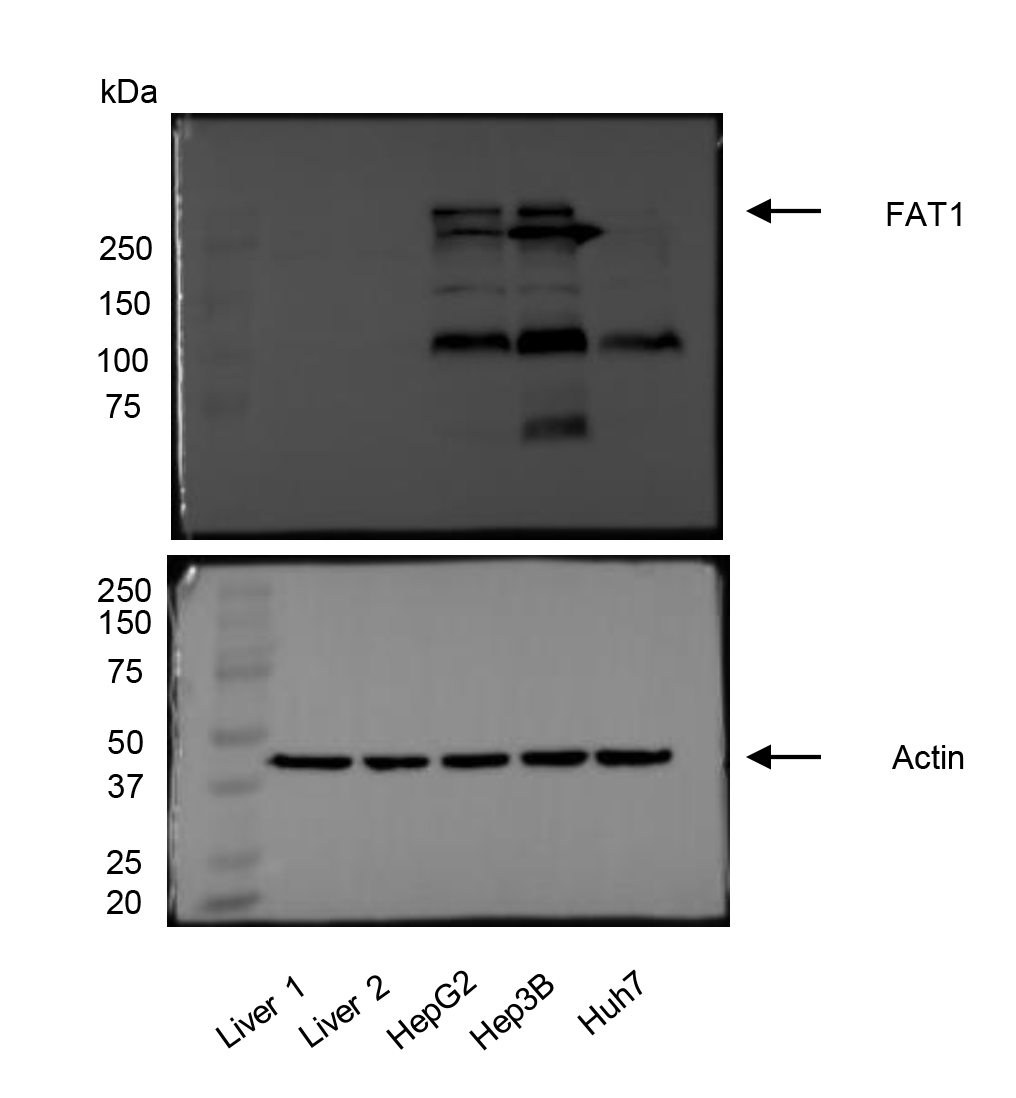
**

**B**

**Fig. S3** Elevated expression of GPC3 and FAT1 in HCC cell lines.

**A)** GPC3 protein levels in two primary liver tissues and HCC cell lines (**Fig. 3C** in the main text).

**B**) FAT1 protein levels from the above-mentioned samples (**Fig. 3D** in the main text).

**
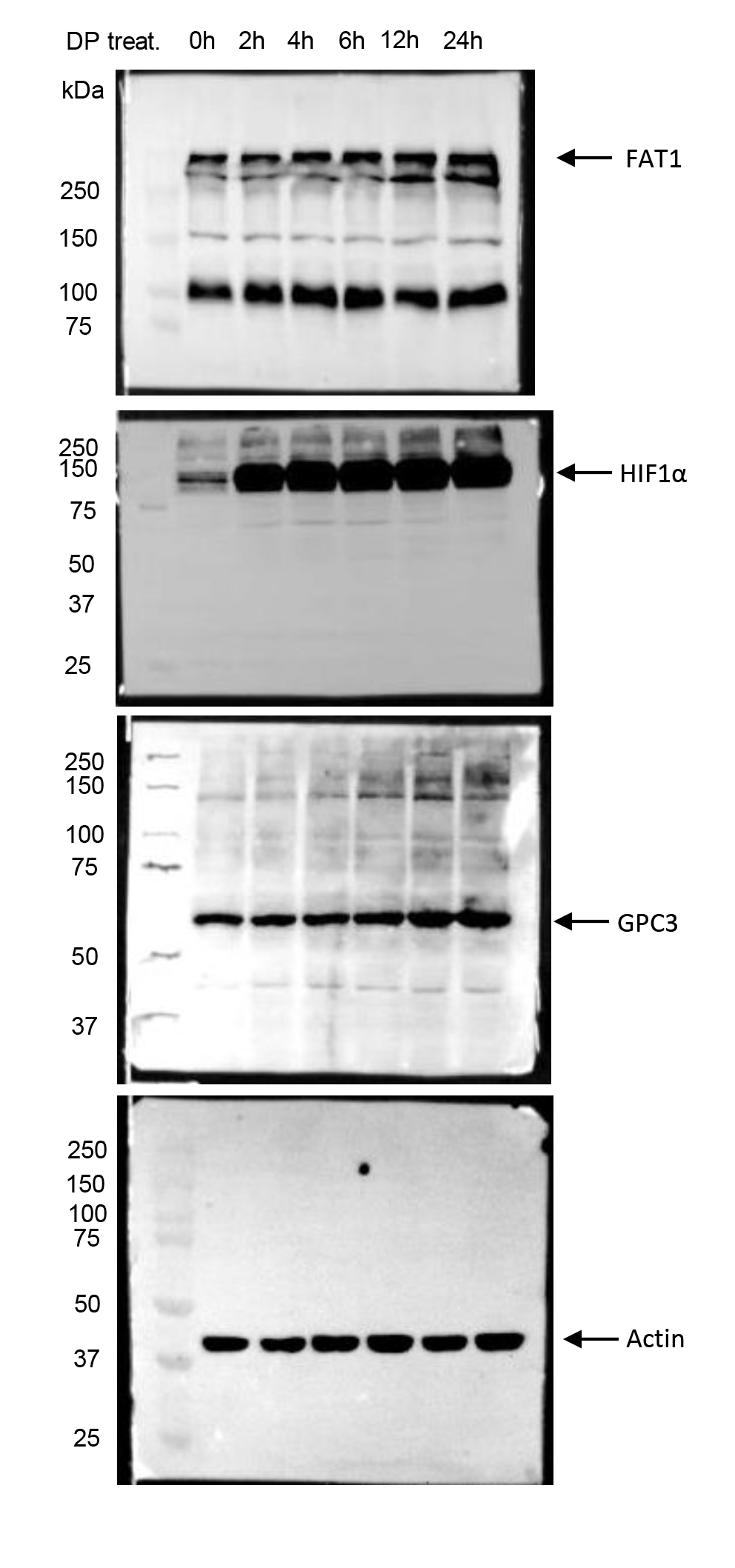
**

**Fig. S4** Western blot analysis of HIF1α, GPC3, and FAT1 protein levels in HepG2 cells after DP (100 µM) treatment (**Fig. 4D** in the main text).

**
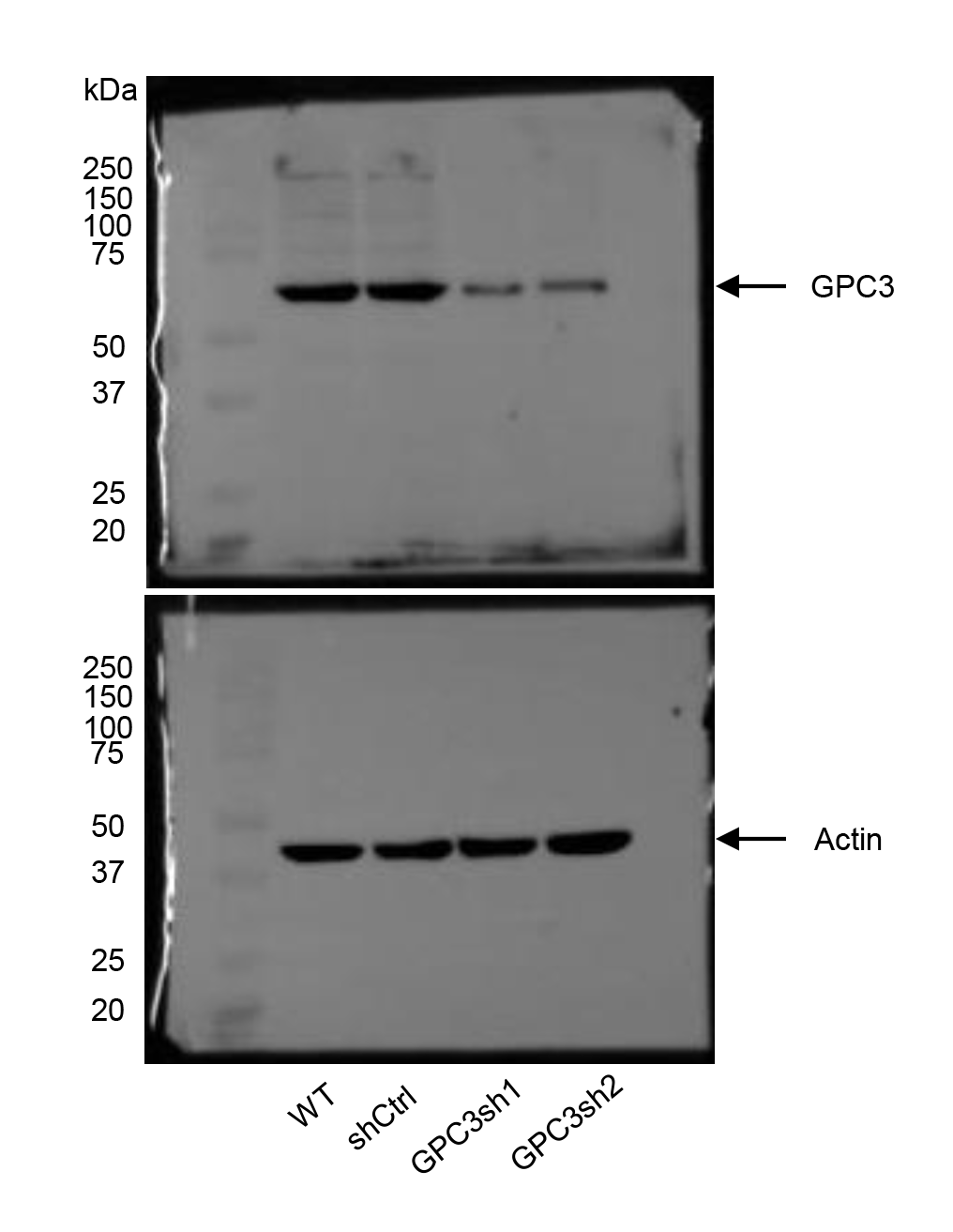
**

**A**

**
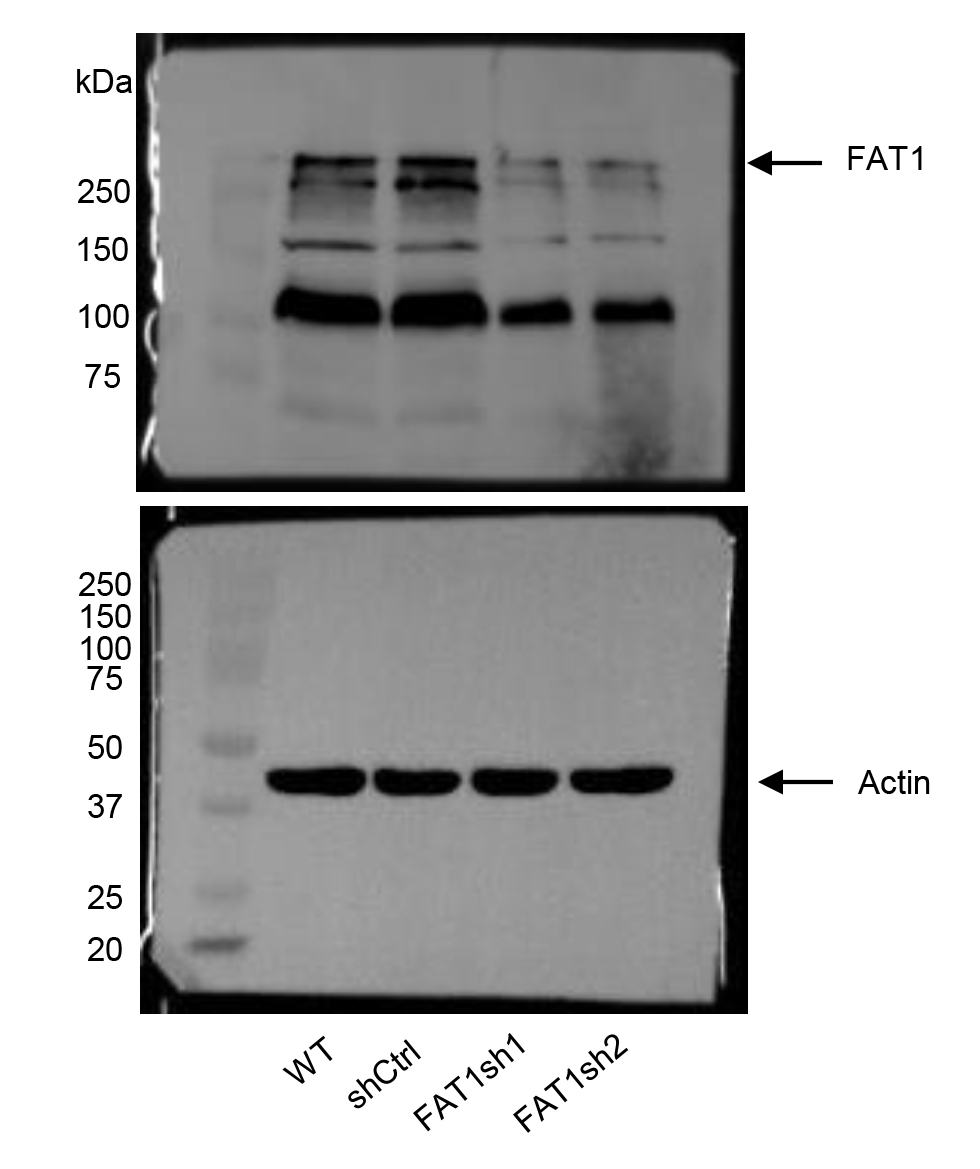
**

**B**

**Fig. S5** Involvement of GPC3 and FAT1 in HCC cell migration.

**A**) Knock-down of GPC3 in Hep3B cells by stable transfection with GPC3 specific shRNA (GPC3sh1 and GPC3sh2) or control shRNA (shCtrl) (**Fig. 5A** in the main text).

**B**) FAT1 knock-down (**Fig. 5B** in the main text).
